# Prospective Associations between Cumulative Average Intake of Flavonoids and Hypertension Risk in the CArdioVascular Disease Association Study (CAVAS)

**DOI:** 10.3390/nu15051186

**Published:** 2023-02-27

**Authors:** Ji-Sook Kong, Yu-Mi Kim, Hye-Won Woo, Min-Ho Shin, Sang-Baek Koh, Hyeon-Chang Kim, Jin-Ho Shin, Mi-Kyung Kim

**Affiliations:** 1 Department of Preventive Medicine, College of Medicine, Hanyang University, Seoul 04763, Republic of Korea; 2 Institute for Health and Society, Hanyang University, Seoul 04763, Republic of Korea; 3 Department of Preventive Medicine, Chonnam National University Medical School, Gwangju 61186, Republic of Korea; 4 Department of Preventive Medicine, Institute of Occupational Medicine, Yonsei Wonju College of Medicine, Wonju 26493, Republic of Korea; 5 Department of Preventive Medicine and Public Health, College of Medicine, Yonsei University, Seoul 03722, Republic of Korea; 6 Department of Internal Medicine, Hanyang University Hospital, Seoul 04763, Republic of Korea

**Keywords:** hypertension, flavonoids, subclasses, obesity, cohort, prospective, Republic of Korea, CAVAS

## Abstract

In this study, we aimed to investigate the prospective associations and their shapes between the dietary intake of total flavonoids and their seven subclasses and hypertension risk in a prospective cohort, the KoGES_CArdioVascular disease Association Study (CAVAS), and to consider obesity status as an additional factor. A total of 10,325 adults aged 40 years and older were enrolled at baseline, and 2159 patients were newly diagnosed with hypertension during a median follow-up of 4.95 years. Cumulative dietary intake was estimated using a repeated food frequency questionnaire. Incidence rate ratios (IRRs) with 95% confidence intervals (CIs) were estimated using modified Poisson models with a robust error estimator. We observed nonlinear inverse associations between total and seven subclasses of flavonoids and hypertension risk, although there was no significant association between total flavonoids and flavones with hypertension risk in the highest quartile. For men, these inverse associations tended to be pronounced in the high BMI group, particularly for anthocyanins and proanthocyanidins [IRR (95% CI) in overweight/obese men: 0.53 (0.42–0.67) for anthocyanins; 0.55 (0.42–0.71) for proanthocyanidins]. Our results suggested that consumption of dietary flavonoids may not be dose-responsive but is inversely associated with hypertension risk, particularly among overweight/obese men.

## 1. Introduction

Hypertension is one of the most important risk factors for all-cause mortality and cardiovascular diseases (CVD) [1,2]. High blood pressure (BP) was attributed to 10.8 million deaths worldwide [3]. Additionally, the prevalence and absolute burden of hypertension are rising globally. Specifically, the global age-standardized prevalence of hypertension was estimated to be 30.1% and 31.9% in women and men, respectively [4]. Therefore, maintaining an optimal BP is a key factor in reducing the burden of diseases related to hypertension. To maintain an optimal BP, certain lifestyle changes are necessary; these changes include adopting a healthy diet and exercising. Overall, these are the cheapest and most sustainable ways to achieve this aim.

Flavonoids are polyphenolic phytochemicals with potential health benefits arising from their antioxidant activities [5]. Additionally, core components of healthy dietary patterns, including the Dietary Approach to Stop Hypertension (DASH) and the Mediterranean diet, such as fruits, vegetables, and legumes, are rich sources of flavonoids [6]. Flavonoids have been intensively studied within the context of their role in the development of non-communicable diseases [6,7]. However, few studies have elucidated the relationship between flavonoids and hypertension; this is especially true when the longitudinal association of various subclasses of flavonoids and hypertension is concerned [8]. In addition to the lack of evidence, some issues should be taken into account in the study of the relationship between total flavonoids and their subclasses and hypertension risk: (1) Differences in the combinations of flavonoids consumed across the globe may result from differences in dietary cultures and their corresponding dietary patterns; this issue is exacerbated by the fact that most previous studies looking into this issue have been conducted in Western populations; (2) Dietary assessments may be confounded by measurement errors, and dietary preferences could be changed over the course of the study. However, only single dietary assessment data were used in these studies; (3) Furthermore, a systematic review of the relationship between flavonoids and vascular function pointed to the possibility that it does not follow a classical linear dose-response association [9]. Thus, the shape of the relationship (linearity or non-linearity) should be tested; (4) Finally, a few previous studies showed that the association of dietary factors with hypertension risk differed according to obesity status [10,11]. In addition, it was reported that flavonoids, such as quercetin, may lower BP in a male rat with diet-induced obesity [12]. However, there is no evidence of an association between flavonoids and hypertension, considering obesity status as a stratum.

We aimed to evaluate the prospective associations, as well as their shapes, between the cumulative average intake of the total flavonoids and its seven subclasses and hypertension risk among adults aged 40 years or older. Additionally, we evaluated whether there were differences in the associations between dietary flavonoids and hypertension risk according to obesity status.

## 2. Materials and Methods

### 2.1. Study Population

The Korean CArdioVascular disease Association Study (CAVAS) was a prospective multicenter cohort study that was part of the population-based Korean Genome and Epidemiology Study (KoGES) consortium [13]. CAVAS was established to investigate the associations of genetic and environmental risk factors with cardiometabolic diseases. Furthermore, this study was a combined cohort of three rural community cohorts: the Multi-Rural Communities Cohort (MRCohort), composed of Yangpyeong, Namwon, and Goryeong counties; the ARIRANG, composed of Wonju and Pyeongchang counties; and the Kangwha cohort. Using multistage cluster sampling, each cohort recruited community-dwelling residents aged ≥40 years. A total of 19,546 participants without CVD and/or cancer participated in the baseline survey between 2005 and 2011 (the MRCohort recruited 9759 participants, the ARIRANG recruited 5942 participants, and the Kangwha cohort recruited 3845 participants from Kangwha) and were followed up every 2–4 years from 2007 to 2017, with 78.2% of them having more than one revisit (Supplementary Figure S1). Interviewers and examiners, who were trained by trainees from the quality control center, collected data in a manner that strictly adhered to standard protocols for questionnaire surveys and examinations. This was necessary to prevent limitations associated with multicenter studies from impacting the validity of the resultant findings.

We excluded participants with the following conditions in the baseline survey: prior physician-diagnosed hypertension and taking antihypertensive medications; systolic BP (SBP) ≥ 140 mmHg or diastolic BP (DBP) ≥ 90 mmHg (n = 8087); missing information on the baseline hypertension identification (n = 39); incomplete (≥10 items blank on the food frequency questionnaire (FFQ)) or implausible (≥ 99.5th or ≤ 0.5th percentile of total energy intake) dietary intake (n = 179); or missing data on covariates (n = 335) including education level, smoking status, regular exercise, alcohol consumption, body mass index (BMI), family history of hypertension, menopausal status (only women), serum creatinine, and diabetes status identification. Apart from these exclusions, participants with serum creatinine >2 mg/dL (n = 5) or prior physician-diagnosed diabetes and taking oral hypoglycemic agents or insulin (n = 578) were also excluded. The final analysis included 10,325 adults (3766 men and 6559 women) (Supplementary Figure S2).

The study was conducted following the principles of the Declaration of Helsinki, and the study protocol was approved by the Institutional Review Boards of Hanyang University, Chonnam University, Keimyung University, Yonsei Wonju College of Medicine, and Yonsei University. All participants provided written informed consent before participating in the study.

### 2.2. Assessments of Dietary Exposures and Covariates

Well-trained interviewers assessed food intake at baseline and revisited every 2–4 years using a validated FFQ comprising 106 food items [14]. Participants were asked to indicate how often 106 foods and beverages were consumed in the preceding year, as well as their average portion size. There were nine categories (“never or rarely” to “3 times/day”) and three portion sizes (mostly 0.5, 1.0, or 1.5 standard portion sizes).

Daily nutrient intake for nutrients other than flavonoids was calculated by weighting frequencies per day and portion sizes for each food item based on the nutrient database in the Seventh Edition of the Korean Food Composition Table [15]. Regarding dietary exposure (flavonoids), a total of 35 flavonoids (seven subclasses: flavonols, flavones, flavanones, flavan-3-ols, anthocyanins, isoflavones, and proanthocyanidins) were estimated based on three databases of the US Department of Agriculture (USDA) (flavonoids [16], isoflavones [17], and proanthocyanidins [18]) and the Phenol-Explorer 3.6 database of the French National Institute for Agricultural Research [19]. If flavonoid content was not available for a specific food, the value for a similar food (same family or similar species belonging to the same genus) was assigned. In the case of foods composed of multiple ingredients, each flavonoid value was calculated from the contents of each flavonoid-containing ingredient.

To reflect healthy dietary patterns, a modified DASH score, after excluding fruits and vegetables and excluding nuts from the nuts or legumes category, was calculated from the following six dietary components [20]: nuts, dairy products except for ice cream, whole grains, sodium, red and processed meats, and sugar-sweetened beverages (SSB). Since fruits, vegetables, and legumes were the main sources of flavonoids, we excluded them. Each component was ranked into quintiles (tertiles for nuts and SSB) and assigned scores between 1 and 5 (or 3). For healthy components, the highest quantile was assigned a score of 5 or 3; for less healthy components, the highest quantile was assigned a score of 1. Finally, we summed up each component score, and the possible overall scores ranged from 6 to 26.

To reduce measurement error in dietary assessment and reflect the long-term diet, we used the cumulative average intake of flavonoids and other nutrients calculated by averaging their intake at baseline and each follow-up survey before the diagnosis of hypertension or at the end of the follow-up [21].

### 2.3. Ascertainment of Outcome: BP Measures and Hypertension Incidence

According to a standard protocol, trained examiners measured BP in the right arm twice and consecutively, with a 5-minute resting period in between, using a standard mercury sphygmomanometer in the MRCohort (Baumanometer, Baumanometer Co., New York, NY, USA) and the ARIRIANG (Baumanometer, Baumanometer Co., USA, and CK-101; Spirit Medical Co., New Taipei City, Taiwan) and an automatic sphygmomanometer in the Kangwha cohort (Dinamap 1846 SX/P, General Electric Co., Boston, MA, USA). When the standard mercury sphygmomanometer was used, the first and fifth Korotkoff sounds were used to measure the SBP and DBP to the nearest 2 mmHg, respectively. If there was a difference of ≥5 mmHg between two consecutive readings, BP was measured again. The arithmetic mean was used for further analysis. However, in the ARIRANG, BP was measured only once before joining the CAVAS (56.2%); thus, for participants in this situation, a single reading was used in the analysis.

At follow-up examinations, participants were asked whether they had been diagnosed with hypertension by a physician and if they had taken any antihypertensive medication between visits. We defined incident cases of hypertension as participants with the following conditions at follow-up: (1) newly diagnosed with hypertension by a physician and taking antihypertensive medications; or (2) measured BP higher than the criteria at follow-up examination (SBP ≥ 140 mmHg or DBP ≥ 90 mmHg), based on the criteria of the Seventh Report of the Joint National Committee on Prevention, Detection, Evaluation, and Treatment of High Blood Pressure [22].

### 2.4. Assessment of Non-Dietary Covariates

Data on demographics (sex, age, and education level) and previously established risk factors for hypertension, such as regular exercise, smoking status, drinking status, family history of hypertension [23,24,25,26], and menopausal status, were collected by trained interviewers using a structured questionnaire. Additionally, anthropometrics was measured during each health examination. Height was measured using a standard stadiometer to the nearest 0.1 cm, and weight was measured using a metric scale in light clothing without shoes to the nearest 0.1 kg. BMI was calculated using the ratio of weight (kg) to height squared (m^2^). Participants were divided into two groups based on their obesity status: normal weight (BMI < 23) and overweight/obese (BMI ≥ 23).

### 2.5. Statistics Analysis

All analyses were conducted separately for men and women. The cumulative average intake of dietary flavonoids and their subclasses were categorized into quartiles (Q). We presented means with standard deviations (SD) for continuous variables and frequencies with percentages for categorical variables to describe the characteristics of the study population. General linear models (GLM) were used to present both age-adjusted estimates and linear trends of covariates by the quartiles of dietary exposure. The mean differences across the quartiles of flavonoids and their subclasses were obtained using Tukey’s multiple comparison test.

We used a modified Poisson regression model with a robust error estimator to estimate incidence rate ratios (IRRs) and 95% confidence intervals (CIs) of hypertension risk by the quartiles of total flavonoids and their seven subclasses (flavonols, flavones, flavanones, flavan-3ols, anthocyanins, isoflavones, and proanthocyanidins) using the lowest quartile (Q1) as reference category [27,28]. We considered three models to examine the risk of hypertension: (1) age-adjusted; (2) multivariable model 1: adjusting for age (years), a higher education level (≥12 years of schooling, yes or no), regular exercise (≥3 times/week and ≥30 min/session, yes or no), smoking (never/past/current smoker for men; non/current smoker for women), current drinkers (yes or no), BMI (kg/m^2^), total energy intake (kcal/day), family history of hypertension (yes or no in the first-degree relatives), menopausal status (yes or no for only women), and baseline BP (SBP and DBP, mmHg); and (3) multivariable model 2, adjusting for all covariates in multivariable model 1 and modified DASH score to consider a possible confounding of healthy dietary patterns [26]. Additionally, to evaluate associations between flavonoid intake from their major food sources and hypertension risk, we selected foods contributing at least 10% of the intake of each flavonoid class and/or at least 10% of the variation (R^2^) from multiple stepwise regression using total intake from all food items as the dependent variable and intake from each food item as the independent variable. Linearity was tested by treating the median cumulative average intake of total flavonoid and their subclasses in each quartile as a continuous variable. To consider a possible curvilinear association suggested by a systematic review of the relationship between flavonoids and vascular function [9], we accounted for the inherent nonlinearity of the fitted categorical model by comparing the deviance difference between the linearity model on 1 degree of freedom (d.f.) and ℓ ordered the categorical model on ℓ − 1 d.f. [29]. The associations between flavonoids and hypertension risk were reanalyzed in each stratum of obesity status (normal and overweight/obese groups). The presence of potential interactions was checked by adding the cross-product term of the flavonoid categories and obesity status group.

To test the robustness of the primary findings, we performed sensitivity analyses in several different ways: by testing the associations (1) after censoring participants who reported a diagnosis of CVD or cancer (533 participants) between visits to minimize the potential effect on the treatment of hypertension; (2) after excluding incident cases of hypertension within the first 2 years to account for the potential reverse causation (n = 9792; n = 3568 for men, n = 6224 for women); (3) using only non-users of antioxidant supplements (vitamin supplements and/or beta-carotene supplements) (n = 8026; n = 3112 for men, n = 4914 for women); and (4) for all 35 individual flavonoids. All statistical analyses were performed using SAS (version 9.4; Cary, NC, USA) and R (version 4.0.0; R Development Core Team, Vienna, Austria). Statistical significance was set at a *p*-value < 0.5, which was considered statistically significant.

## 3. Results

A total of 2159 cases of hypertension were identified during the 53,678 people’s follow-ups (Supplementary Table S1). The mean age at baseline was 56.9 years (SD, 9.73), and 36.5% of the population were men (n = 3766). The cumulative average intake of total dietary flavonoids was 176 mg/day (SD, 164), and the highest proportion of seven subclasses of flavonoids was found in proanthocyanidins (33.1% for men and 37.3% for women), followed by flavan-3-ols, isoflavones, flavanols, flavanones, anthocyanins, and flavones (Supplementary Table S2). The baseline characteristics and cumulative dietary intakes of the total and seven subclasses of flavonoids are presented by the quartiles (Q) of total flavonoid intake (Table 1). Men and women in the highest quartile (Q4) were younger, highly educated, exercised regularly, never smoked, had a higher BMI, and had a higher rate of self-reported family history of hypertension. They tended to have a higher intake of energy and all flavonoid subclasses and higher scores on the modified DASH.

Table 2 presents the association between the intake of flavonoids and hypertension risk. Flavonoids tended to be inversely associated with hypertension after adjusting for multiple covariates (multivariable model 1), although total flavonoids and flavones were not significant in Q4 in both men and women. The inverse association of six flavonoids, other than flavones, seemed to be non-linear L-shaped, although the associations of anthocyanins and proanthocyanidins for both men and women and flavanones for women were statistically linear (*P*_linearity_ < 0.05, but not significant at 0.05/16 tests = 0.0031). The associations and their shapes, after additional adjustment for the modified DASH score (multivariable model 2), remained robust; therefore, the multivariable model without.

The associations between dietary flavonoid intake from their major food sources and hypertension risk are shown in Table 3. A few foods explained a large proportion of the total variation; for example, green tea explained about 68% of the variation in total flavonoid intake and almost all variations in flavan-3-ols in both men and women. The inverse associations remained robust. Moreover, in both men and women, even for total flavonoids and flavones which were not associated with hypertension risk in Table 2, the associations of those from individual major food sources with hypertension risk were consistently inverse, except for flavones from pickled vegetables in salt (*Baechu-kimchi*). Flavones from pickled vegetables were not associated with hypertension risk in men and were positively associated with hypertension risk in women.

**Figure 1:**
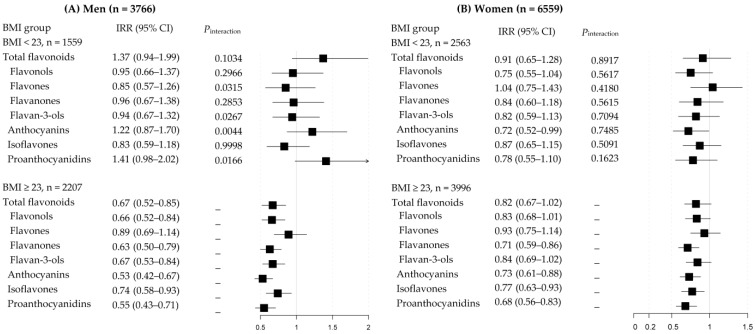
Incident Rate Ratios (IRRs) and 95% Confidence Intervals (CIs) of hypertension in the highest quartile of flavonoids (vs. the lowest quartile) based on BMI group; (**A**) men (**B**) women. The multivariable model was adjusted for age (years), a higher education level (≥12 schooling years), regular exercise (≥3 times/week and ≥30 min/session), smoking (current/past/non-smokers for men and current/non-smokers for women), current drinkers (yes or no), body mass index (BMI), total energy intake (kcal/d), family history of hypertension (yes or no), menopausal status (yes or no for only women), and the baseline blood pressures. *P*-interaction was tested by adding the cross-product term of quartiles of flavonoid and BMI groups (≥ 23.0 kg/m^2^, yes/no).

Figure 1 presents the associations of flavonoids with hypertension risk in each obesity status stratum. The beneficial associations for hypertension tended to be stronger in overweight/obese men (BMI, ≥23.0 kg/m^2^) [IRR (95% CI) in overweight/obese men and *p*-value for interaction by BMI groups: 0.67 (0.53–0.84) and *P_inter_* = 0.0267 for flavan-3-ols; 0.53 (0.42–0.67) and *P_inter_* = 0.0044 for anthocyanins; 0.55 (0.42–0.71) and *P_inter_* = 0.0166 for proanthocyanidins]. In women, there were no differences in the association of flavonoids with hypertension risk according to obesity status.

These associations remained consistent in three sensitivity analyses: censoring participants who developed CVD and cancer during follow-up, excluding participants who reported hypertension within the first two years after baseline, and including only non-users of antioxidant supplements (77.7%) (Supplementary Table S3). The associations between the 35 individual flavonoids and the risk of hypertension were similar to the findings of their corresponding subclasses (Supplementary Table S4).

## 4. Discussion

In this prospective cohort study with 10,325, we found an association between various subclasses of flavonoids and lowered hypertension risk. However, these associations were not significant in the highest quartile of total flavonoids and flavones (reverse J-shapes). The shapes of the associations for flavonoids other than total flavonoids and flavones appeared to be non-linear and L-shaped, with plateaus from moderate intakes. Inverse associations were more pronounced in men with high BMI.

The mean total dietary flavonoid intake was 176 mg/day (SD, 164 mg/day) and higher in men [161 mg/day (SD, 142) in men and 185 mg/day (SD, 175) in women]. Flavonoid intake values across studies may not be directly comparable because of the different flavonoid assessment tools, but the mean total flavonoid intake worldwide ranges between 150 and 600 mg/day [20]. In the present study, the highest contribution to total flavonoids in both men and women was proanthocyanidins, followed by flavan-3-ols, which is consistent with a previous report that they are the most abundant sources of flavonoids in East Asian countries such as China and South Korea [20]. This can be explained by the fact that flavonoids are ubiquitous in plants as secondary metabolites but are particularly abundant in fruits, vegetables, legumes, and beverages such as green tea [20].

In the present study, we found a reverse J-shaped association for total flavonoids (not significant in Q4) and L-shaped associations for most of its subclasses. Therefore, the inverse associations of the subclasses were stronger than those of the total flavonoids. To date, few prospective studies have examined the association between hypertension and total flavonoid intake and/or its subclasses. However, (1) in the previous literature review on the association between flavonoids and CVD risk [30], it was suggested that there might be a plateau in the trend; specifically, higher intakes might afford no added benefit, and our findings are consistent with this suggestion. In addition, (2) previous studies also found that the effects of some subclasses were similar to or exceeded those of the total flavonoids [31,32,33,34], like our findings: In middle-aged French women, the association of total flavonoids with hypertension risk showed a similar magnitude to the inverse associations of flavonols, anthocyanins, and proanthocyanidin polymers [32]. In pooled analyses of three prospective studies in middle-aged and older US adults, anthocyanin intake and flavan-3-ol compounds were inversely associated with the risk of hypertension [33], whereas total flavonoids were not associated with hypertension incidence. Among Australian women in two different life stages, individual flavonoid subclasses were different but inversely associated with a lower risk of hypertension [31]. They showed an inverse association of flavones, isoflavones, and flavanones with hypertension risk among middle-aged women and flavanols among reproductive-aged women [31], whereas there was no association between total flavonoids and hypertension incidence. These less beneficial findings for total flavonoids but strongly beneficial findings for different flavonoids may reflect various dietary cultures across populations in which harmful or beneficial substances other than flavonoids can be mixed. Flavones from *Baechu-kimchi* were found to have no or a positive association with hypertension risk in this study. *Baechu-kimchi* is the most commonly consumed side dish in Korea and is a sodium-rich food [35]. Increased sodium intake might lead to sodium-dependent oxidative stress by abolishing local nitric oxide activity and increasing microvascular reactive oxygen species (ROS) levels [36]. Although in our data, additionally adjusting for dietary sodium intake did not change the associations, it is necessary to consider sodium intake using a more accurate estimation method such as 24-h urine data [37].

Although there is no single mechanism or individual flavonoids to prevent hypertension and CVD [6], the main biological activities of flavonoids in the etiology of hypertension are enhanced endothelial function, antioxidant activity, anti-inflammatory properties, and antithrombotic activities [6,38]. In terms of individual flavonoid subclasses, the bioavailability of flavonoids depends on their composition, the total number of hydroxyl groups, and the substitution of functional groups [5]. Among flavonoids, anthocyanins, and proanthocyanidins, which are the most numerous and widely distributed pigments in plants, have been reported to have health benefits for BP owing to their potential antioxidant activities [39]. Flavanones are found in high concentrations in citrus fruit and mainly improve endothelial function by enhancing blood flow, increasing endothelial nitric oxide synthase activity, and inhibiting platelet function [38,40,41].

In addition, we found more pronounced beneficial associations with flavonoids in overweight and obese men but not in normal-weight men. There was no difference in obesity status among women. Previously, it has been demonstrated that the risk of obesity-associated hypertension has sex differences [42]. Our findings in obese men may be explained by the suppression of over-activation of the sympathetic nervous system by flavonoids. Previous studies have reported that sympathetic nerve activation (SNA), which is a significant contributing factor to hypertension, can be activated, particularly in men with high BMI [43], and the injection of antioxidants directly results in decreased SNA and BP [44]. Although some previous studies have suggested differences in age group [33] or sex [8], we did not find any other differences by covariates in the effect modification analyses after stratification of all covariates (data not shown).

This study had several limitations. Firstly, the present study used a prospective cohort design and considered the cumulative average dietary intake of flavonoids. However, we could not conclude the causal relationship between flavonoids and hypertension risk (1) because unmeasured confounding factors may still exist and (2) because there is a possibility that participants who had normal BP at baseline but developed prehypertension during the follow-up period may have changed their dietary habits to increase flavonoids intake. However, this possibility suggested that the association between flavonoid intake and hypertension in the present study may be underestimated. Furthermore, (3) to provide more robust evidence on the association between flavonoids and hypertension risk, further studies are needed using new emerging data analysis methods, including counterfactual models for causal inference and accumulating evidence from large-scale cohorts and randomized trials. Second, the flavonoid content in foods can be influenced by the cooking process, geographic area, seasonality, and food growth, but we could not consider these factors. Although this may attenuate the association, it is unlikely to cause a differential misclassification of exposure. Third, our FFQ did not include enough flavonoid-rich foods and seasonings, such as herbs [31], and some foods, such as berries and herbs, have recently gained popularity in Korea [14]. This may lead to an underestimation or misclassification of flavonoid intake. Fourth, BP measurement protocols in the three cohorts were different at the beginning of the cohort before joining CAVAS, as we mentioned above. Therefore, we conducted a pooled analysis with findings from separate analyses of each of the three cohorts and confirmed similar results. Fifth, although we considered comprehensive confounders such as baseline BP and DASH score, it is difficult to disentangle the unique effects of flavonoids from other dietary compounds such as vitamin C, potassium, folate, and magnesium, which are rich sources of flavonoids [6,8]. Lastly, dietary flavonoid intake does not reflect inter- and intra-individual variations such as absorption and metabolism; therefore, their association with health outcomes should be interpreted with caution [16]. Nevertheless, the present study had several strengths, such as its prospective design, large sample size, and repeated reliable assessments of individual diets.

In conclusion, our findings indicated that dietary flavonoid subclasses, as well as total dietary flavonoid intake, may be beneficial for hypertension risk in a non-linear reverse J- or L-shaped manner in both men and women. In men, these favorable associations may be predominant in the overweight/obese group.

## Figures and Tables

**Table 1:** Age-adjusted characteristics of the study participants by dietary intake of total flavonoids (n = 10,325) ^a^.

Characteristics	Men (n = 3766)				*P* diff ^b^	Women (n = 6559)				*P* diff
	Q1	Q2	Q3	Q4		Q1	Q2	Q3	Q4	
N (%)	941	942	942	941	_	1639	1640	1640	1640	_
No. cases/ person-year	242/4392	199/4992	186/5078	227/4908	_	381/7568	306/8966	315/9164	303/8610	_
Age, y	61.2 ± 0.31	59.2 ± 0.31	58.1 ± 0.31	55.6 ± 0.31	<0.0001	60.9 ± 0.22	56.6 ± 0.22	54.1 ± 0.22	52.1 ± 0.22	<0.0001
Higher education ^c^	27.2	36.3	40.0	49.9	<0.0001	21.0	22.9	29.0	37.7	<0.0001
Regular exercise ^d^	11.9	17.4	22.2	28.4	<0.0001	13.7	17.3	24.2	32.1	<0.0001
Current smoker										
Never-smoker	23.8	22.5	30.0	32.9	<0.0001	93.7	96.7	97.1	95.9	<0.0001
Past smoker	31.8	35.2	33.0	35.6	0.2608	1.60	1.28	0.84	1.47	0.2536
Current smoker	44.4	42.2	37.0	31.6	<0.0001	4.68	1.99	2.05	2.67	<0.0001
Menopausal status	_	_	_	_	_	67.0	71.0	72.9	69.4	<0.0001
Family history of hypertension	13.8	13.2	13.1	15.6	0.3835	17.0	18.9	20.8	23.4	<0.0001
Current drinker	63.1	62.8	61.0	63.5	0.6620	30.6	29.5	30.1	31.1	0.7747
Alcohol consumption, ml/d	28.3 ± 1.80	26.4 ± 1.78	25.7 ± 1.78	26.7 ± 1.80	0.7836	2.73 ± 0.28	1.98 ± 0.27	2.22 ± 0.27	2.75 ± 0.28	0.1143
Body Mass index, kg/m^2^	23.4 ± 0.09	23.6 ± 0.09	23.7 ± 0.09	24.0 ± 0.09	<0.0001	23.8 ± 0.08	24.0 ± 0.08	24.0 ± 0.08	24.2 ± 0.08	0.0377
Total energy intake, kcal/d	1421 ± 12.7	1644 ± 12.5	1784 ± 12.5	1949 ± 12.7	<0.0001	1254 ± 9.38	1451 ± 9.05	1587 ± 9.09	1744 ± 9.24	<0.0001
Modified DASH scores ^e^	16.6 ± 0.10	17.3 ± 0.10	17.3 ± 0.10	17.7 ± 0.10	<0.0001	16.9 ± 0.08	17.3 ± 0.08	17.6 ± 0.08	18.0 ± 0.08	<0.0001
Total flavonoids, mg/d	45.7 ± 2.72	92.6 ± 2.69	156 ± 2.69	348 ± 2.72	_	51.1 ± 2.75	103 ± 2.65	177 ± 2.66	407 ± 2.71	<0.0001
Flavonols	10.0 ± 0.38	14.6 ± 0.38	19.1 ± 0.38	28.8 ± 0.38	<0.0001	9.14 ± 0.31	14.5 ± 0.29	18.6 ± 0.30	30.0 ± 0.30	<0.0001
Flavones	0.95 ± 0.03	1.40 ± 0.03	1.90 ± 0.03	2.66 ± 0.03	<0.0001	0.96 ± 0.03	1.54 ± 0.03	2.03 ± 0.03	3.12 ± 0.03	<0.0001
Flavanones	2.31 ± 0.26	4.64 ± 0.26	8.26 ± 0.26	13.2 ± 0.26	<0.0001	3.21 ± 0.25	6.73 ± 0.24	11.2 ± 0.25	19.4 ± 0.25	<0.0001
Flavan-3-ols	6.68 ± 2.54	16.5 ± 2.51	38.9 ± 2.51	161 ± 2.54	<0.0001	8.63 ± 2.47	17.0 ± 2.38	40.8 ± 2.39	174 ± 2.43	<0.0001
Anthocyanins	1.72 ± 0.25	4.44 ± 0.25	8.52 ± 0.25	14.3 ± 0.25	<0.0001	2.17 ± 0.25	5.89 ± 0.24	10.8 ± 0.24	19.8 ± 0.24	<0.0001
Isofavones	10.3 ± 0.48	19.1 ± 0.48	23.0 ± 0.48	26.3 ± 0.48	<0.0001	10.0 ± 0.36	17.5 ± 0.34	22.3 ± 0.35	25.2 ± 0.35	<0.0001
Proanthocyanins	13.7 ± 1.31	32.1 ± 1.30	56.3 ± 1.30	102 ± 1.32	<0.0001	17.0 ± 1.28	40.1 ± 1.24	71.4 ± 1.24	136 ± 1.26	<0.0001

^a^ Values were adjusted for age and expressed as mean ± standard error for continuous variables and percentages for categorical variables. ^b^ *p*-values for differences were determined by the General Linear Model. ^c^ Higher education level (≥12 schooling years of education). ^d^ Regular exercise (≥3 times/week and ≥30 min/session). ^e^ Modified Dietary Approaches to Stop Hypertension (DASH) scores were calculated based on six out of eight components after excluding fruits and vegetables and modifying the nuts/legumes into nuts without legumes.

**Table 2:** Incident Rate Ratios (IRRs) and 95% Confidence Intervals (CIs) of hypertension by quartiles of dietary intakes of flavonoids and its subclasses (n = 10,325).

Flavonoids, mg/d	Men (n = 3766)						Women (n = 6559)					
	Q1	Q2	Q3	Q4	*P*_linear_^a^	*P*_non-linear_^b^	Q1	Q2	Q3	Q4	*P*_linear_	*P*_non-linear_
**Total flavonoids**												
Median (min, max)	46.7 (3.52, 69.3)	91.9 (69.3, 118)	153 (118, 206)	304 (206, 1929)			50.1 (1.05, 75.7)	102 (75.8, 133)	175 (133, 236)	345 (236, 2485)		
Age-adjusted model	1 (ref)	0.74 (0.62–0.89)	0.70 (0.58–0.84)	0.91 (0.76–1.09)	0.9233	<0.0001	1 (ref)	0.80 (0.69–0.93)	0.88 (0.75–1.02)	0.99 (0.84–1.16)	0.3674	<0.0001
Multivariable model 1 ^c^	1 (ref)	0.70 (0.58–0.84)	0.70 (0.57–0.85)	0.84 (0.68–1.03)	0.8302	<0.0001	1 (ref)	0.73 (0.63–0.85)	0.80 (0.68–0.93)	0.86 (0.72–1.03)	0.7528	0.0002
Multivariable model 2 ^d^	1 (ref)	0.70 (0.58–0.85)	0.70 (0.58–0.86)	0.85 (0.69–1.04)	0.8853	0.0001	1 (ref)	0.74 (0.63–0.86)	0.80 (0.68–0.94)	0.87 (0.72–1.04)	0.8415	0.0002
**Flavonols**												
Median (min, max)	7.09 (0.57, 9.81)	12.4 (9.83, 15.0)	18.1 (15, 22.3)	29.6 (22.4, 246)			6.33 (0.11, 8.98)	11.6 (8.99, 14.5)	17.7 (14.5, 22.6)	31.4 (22.6, 219)		
Age-adjusted model	1 (ref)	0.68 (0.56–0.81)	0.76 (0.63–0.91)	0.85 (0.71–1.01)	0.4899	<0.0001	1 (ref)	0.78 (0.68–0.91)	0.77 (0.66–0.90)	0.92 (0.79–1.08)	0.8456	<0.0001
Multivariable model 1	1 (ref)	0.63 (0.53–0.76)	0.69 (0.57–0.84)	0.75 (0.61–0.92)	0.1165	<0.0001	1 (ref)	0.73 (0.63–0.84)	0.70 (0.60–0.82)	0.81 (0.69–0.96)	0.2043	<0.0001
Multivariable model 2	1 (ref)	0.63 (0.52–0.75)	0.68 (0.56–0.82)	0.73 (0.59–0.89)	0.0671	<0.0001	1 (ref)	0.72 (0.62–0.84)	0.70 (0.59–0.81)	0.80 (0.68–0.95)	0.1692	<0.0001
**Flavones**												
Median (min, max)	0.7 (0, 0.97)	1.22 (0.97, 1.49)	1.78 (1.49, 2.16)	2.86 (2.16, 11.6)			0.70 (0.01, 1.01)	1.30 (1.02, 1.60)	1.96 (1.60, 2.43)	3.27 (2.43, 18.3)		
Age-adjusted model	1 (ref)	0.86 (0.72–1.03)	0.72 (0.59–0.87)	0.99 (0.82–1.19)	0.9740	0.0002	1 (ref)	0.93 (0.80–1.08)	0.85 (0.73–0.99)	1.07 (0.92–1.26)	0.3307	0.0001
Multivariable model 1	1 (ref)	0.78 (0.65–0.93)	0.66 (0.55–0.81)	0.91 (0.73–1.12)	0.6525	<0.0001	1 (ref)	0.91 (0.78–1.05)	0.78 (0.67–0.92)	0.96 (0.80–1.14)	0.7084	0.0037
Multivariable model 2	1 (ref)	0.77 (0.64–0.93)	0.66 (0.54–0.80)	0.89 (0.72–1.10)	0.5638	<0.0001	1 (ref)	0.91 (0.78–1.05)	0.78 (0.66–0.91)	0.95 (0.80–1.14)	0.6656	0.0038
**Flavanones**												
Median (min, max)	0.77 (0, 1.82)	3.11 (1.82, 4.58)	6.47 (4.58, 9.21)	14.3 (9.21, 133)			1.25 (0, 2.95)	4.7 (2.95, 6.79)	9.3 (6.79, 13.3)	19.7 (13.3, 192)		
Age-adjusted model	1 (ref)	0.69 (0.58–0.82)	0.60 (0.50–0.72)	0.74 (0.61–0.89)	0.0432	<0.0001	1 (ref)	0.80 (0.69–0.93)	0.81 (0.69–0.94)	0.83 (0.71–0.98)	0.1246	0.0001
Multivariable model 1	1 (ref)	0.66 (0.55–0.78)	0.56 (0.47–0.68)	0.72 (0.59–0.87)	0.0581	<0.0001	1 (ref)	0.77 (0.67–0.90)	0.76 (0.65–0.89)	0.74 (0.63–0.87)	0.0054	0.0061
Multivariable model 2	1 (ref)	0.66 (0.55–0.79)	0.57 (0.47–0.69)	0.72 (0.59–0.88)	0.0605	<0.0001	1 (ref)	0.78 (0.67–0.90)	0.76 (0.66–0.89)	0.75 (0.63–0.88)	0.0064	0.0069
**Flavan-3ols**												
Median (min, max)	3.26 (0, 6.35)	10.4 (6.36, 17.4)	30.7 (17.4, 52.7)	132 (52.8, 1755)			3.61 (0, 7.05)	11.5 (7.06, 18.5)	31.2 (18.5, 52.6)	135 (52.6, 1808)		
Age-adjusted model	1 (ref)	0.65 (0.54–0.78)	0.72 (0.60–0.86)	0.83 (0.69–0.99)	0.7922	<0.0001	1 (ref)	0.82 (0.71–0.95)	0.92 (0.79–1.07)	0.89 (0.75–1.04)	0.6569	0.0001
Multivariable model 1	1 (ref)	0.64 (0.53–0.77)	0.70 (0.58–0.85)	0.76 (0.63–0.92)	0.6804	<0.0001	1 (ref)	0.82 (0.70–0.95)	0.88 (0.75–1.03)	0.84 (0.72–0.99)	0.3449	0.0300
Multivariable model 2	1 (ref)	0.64 (0.53–0.77)	0.71 (0.59–0.86)	0.76 (0.63–0.92)	0.6851	<0.0001	1 (ref)	0.82 (0.71–0.95)	0.88 (0.75–1.03)	0.85 (0.72–1.00)	0.3745	0.0300
**Anthocyanins**												
Median (min, max)	0.86 (0, 1.9)	3.07 (1.90, 4.60)	6.38 (4.6, 9.01)	14.7 (9.01, 150)			1.25 (0, 2.63)	4.20 (2.63, 6.19)	8.57 (6.19, 12.7)	19.8 (12.7, 225)		
Age-adjusted model	1 (ref)	0.69 (0.58–0.82)	0.62 (0.52–0.75)	0.70 (0.58–0.84)	0.0112	<0.0001	1 (ref)	0.76 (0.65–0.88)	0.71 (0.61–0.83)	0.82 (0.70–0.95)	0.1371	<0.0001
Multivariable model 1	1 (ref)	0.69 (0.58–0.82)	0.61 (0.50–0.73)	0.70 (0.57–0.85)	0.0271	<0.0001	1 (ref)	0.76 (0.66–0.88)	0.70 (0.60–0.82)	0.73 (0.62–0.86)	0.0047	0.0003
Multivariable model 2	1 (ref)	0.69 (0.58–0.82)	0.61 (0.51–0.74)	0.70 (0.57–0.86)	0.0279	<0.0001	1 (ref)	0.76 (0.66–0.89)	0.70 (0.60–0.82)	0.73 (0.62–0.86)	0.0053	0.0003
**Isoflavones**												
Median (min, max)	5.86 (0.25, 8.56)	11.7 (8.58, 15.1)	19.8 (15.1, 25.9)	36.8 (26.0, 157)			5.59 (0, 8.3)	11.1 (8.31, 14.2)	18.7 (14.2, 25.0)	35.5 (25.0, 191)		
Age-adjusted model	1 (ref)	0.74 (0.62–0.89)	0.62 (0.52–0.74)	0.80 (0.67–0.96)	0.1167	<0.0001	1 (ref)	0.76 (0.66–0.89)	0.71 (0.61–0.82)	0.90 (0.78–1.03)	0.6540	<0.0001
Multivariable model 1	1 (ref)	0.76 (0.63–0.92)	0.61 (0.51–0.74)	0.77 (0.64–0.94)	0.0807	<0.0001	1 (ref)	0.75 (0.64–0.87)	0.67 (0.58–0.79)	0.80 (0.68–0.94)	0.1141	<0.0001
Multivariable model 2	1 (ref)	0.77 (0.63–0.92)	0.62 (0.51–0.75)	0.78 (0.64–0.95)	0.1328	<0.0001	1 (ref)	0.75 (0.64–0.87)	0.68 (0.58–0.79)	0.80 (0.68–0.94)	0.1390	<0.0001
**Proanthocyanidins**												
Median (min, max)	11.5 (0, 19.7)	27.4 (19.7, 36.5)	48.7 (36.6, 64.2)	95.4 (64.3, 641)			14.8 (0, 24.3)	34.4 (24.3, 46.3)	62.8 (46.4, 85.6)	126 (85.8, 836)		
Age-adjusted model	1 (ref)	0.85 (0.71–1.01)	0.78 (0.65–0.93)	0.79 (0.66–0.96)	0.0400	0.0750	1 (ref)	0.78 (0.67–0.91)	0.77 (0.66–0.89)	0.80 (0.68–0.94)	0.0493	<0.0001
Multivariable model 1	1 (ref)	0.79 (0.66–0.94)	0.73 (0.61–0.89)	0.74 (0.60–0.92)	0.0342	0.0270	1 (ref)	0.78 (0.67–0.90)	0.69 (0.59–0.81)	0.70 (0.59–0.84)	0.0012	0.0012
Multivariable model 2	1 (ref)	0.80 (0.66–0.96)	0.74 (0.61–0.90)	0.76 (0.61–0.94)	0.0531	0.0380	1 (ref)	0.78 (0.67–0.91)	0.70 (0.60–0.82)	0.71 (0.59–0.85)	0.0020	0.0014

^a^*p*-values for linear trends were obtained by treating the median value of each group as a continuous variable. ^b^*p*-values for non-linear trends were obtained by comparing the deviance difference between the linear trend model on 1 degree of freedom (d.f.) and ℓ ordered categorical model on ℓ− 1 d.f. ^c^ Multivariable model 1 was adjusted for age (years), higher education level (≥12 years of schooling), regular exercise (≥3 times/week and ≥30 min/session), smoking (current/past/non-smokers for men and current/non-smokers for women), current drinkers (yes or no), body mass index (BMI), total energy intake (kcal/d), family history of hypertension (yes or no), menopausal status (yes or no for only women) and baseline blood pressures. ^d^ Multivariable model 2 was adjusted for all covariates in multivariable model 1, and modified DASH scores were calculated based on six out of eight components after excluding fruits and vegetables and modifying the ‘Nuts/legumes’ into ‘Nuts’ without legumes.

**Table 3:** Incident Rate Ratios (IRRs) and 95% Confidence Intervals (CIs) of hypertension by quartiles of the dietary flavonoids and its subclasses from their major food sources (n = 10,325) ^a^.

Food Item	Contribution to Intake (%) ^b^	Percentage of the Variation ^c^	Men (n = 3766)			*P*_linear_^d^	*P*_non-linear_^e^	Contribution to Intake (%)	Percentage of the Variation	Women (n = 6559)			*P*_linear_	*P*_non-linear_
			Q2	Q3	Q4					Q2	Q3	Q4		
**Total flavonoids from **														
Green tea	19.8	68.6	0.59 (0.48–0.73)	0.67 (0.56–0.79)	0.75 (0.63–0.89)	0.2261	<0.0001	17.7	67.1	0.66 (0.55–0.79)	0.87 (0.76–0.99)	0.80 (0.69–0.92)	0.0723	<0.0001
Apple/apple juice	11.5	17.0	0.72 (0.60–0.86)	0.66 (0.55–0.80)	0.63 (0.52–0.76)	0.0005	0.0004	14.2	19.9	0.82 (0.71–0.94)	0.62 (0.53–0.72)	0.72 (0.62–0.85)	0.0055	<0.0001
Grapes/grape juice	11.1	7.45	0.63 (0.53–0.75)	0.60 (0.50–0.72)	0.64 (0.52–0.77)	0.0087	<0.0001	13.3	7.89	0.81 (0.70–0.94)	0.69 (0.59–0.80)	0.67 (0.57–0.78)	<0.0001	0.0038
**Flavonols from**														
Lettuce	17.2	13.2	0.90 (0.75–1.08)	0.65 (0.53–0.79)	0.78 (0.64–0.94)	0.0145	0.0011	18.2	21.1	0.82 (0.71–0.95)	0.67 (0.57–0.78)	0.79 (0.68–0.92)	0.0215	<0.0001
Radish Kimchi	11.4	5.32	1.01 (0.84–1.22)	0.80 (0.67–0.97)	0.83 (0.68–1.00)	0.0192	0.1190	7.88	3.82	0.75 (0.64–0.87)	0.69 (0.59–0.80)	0.76 (0.65–0.88)	0.0667	<0.0001
Other green vegetables ^f^	9.73	49.6	0.51 (0.43–0.60)	0.53 (0.44–0.64)	0.68 (0.57–0.82)	0.2638	<0.0001	12.0	44.5	0.76 (0.66–0.88)	0.59 (0.51–0.69)	0.71 (0.61–0.83)	0.0119	<0.0001
Green tea	8.07	9.29	0.59 (0.48–0.73)	0.67 (0.56–0.79)	0.75 (0.63–0.89)	0.2261	<0.0001	8.06	12.9	0.66 (0.55–0.79)	0.87 (0.76–0.99)	0.80 (0.69–0.92)	0.0723	<0.0001
**Flavones from**														
Green pepper	18.9	38.1	0.79 (0.66–0.95)	0.76 (0.63–0.91)	0.83 (0.68–1.01)	0.3294	0.0113	18.9	39.3	0.77 (0.67–0.89)	0.54 (0.46–0.63)	0.84 (0.73–0.97)	0.2518	<0.0001
*Baechu-kimchi*	16.8	0.97	1.03 (0.86–1.22)	0.45 (0.36–0.57)	1.16 (0.99–1.37)	0.8829	<0.0001	14.4	0.71	0.93 (0.81–1.08)	0.54 (0.47–0.64)	1.25 (1.08–1.45)	0.9591	<0.0001
Tangerine	13.8	11.9	0.71 (0.59–0.85)	0.56 (0.47–0.67)	0.63 (0.53–0.76)	<0.0001	<0.0001	18.2	25.9	0.68 (0.59–0.79)	0.67 (0.57–0.77)	0.79 (0.68–0.93)	0.0900	<0.0001
Orange/orange juice	6.12	25.2	0.56 (0.41–0.75)	0.68 (0.57–0.80)	0.70 (0.58–0.83)	0.0053	<0.0001	7.80	15.5	0.63 (0.52–0.75)	0.77 (0.67–0.88)	0.73 (0.63–0.85)	0.0084	<0.0001
**Flavanones from**														
Tangerine	48.3	25.8	0.71 (0.59–0.85)	0.56 (0.47–0.67)	0.63 (0.53–0.76)	<0.0001	<0.0001	50.6	34.2	0.68 (0.59–0.79)	0.67 (0.58–0.78)	0.79 (0.67–0.92)	0.0705	<0.0001
Grapes/grape juice	26.7	9.94	0.63 (0.53–0.75)	0.60 (0.50–0.72)	0.64 (0.52–0.77)	0.0087	<0.0001	26.0	9.25	0.81 (0.70–0.94)	0.69 (0.59–0.80)	0.67 (0.58–0.78)	<0.0001	0.0039
Orange/orange juice	18.6	64.3	0.56 (0.41–0.75)	0.68 (0.57–0.80)	0.70 (0.58–0.83)	0.0053	<0.0001	19.2	56.5	0.62 (0.52–0.75)	0.77 (0.67–0.88)	0.73 (0.63–0.85)	0.0088	<0.0001
**Flavan-3-ols from**														
Green tea	46.7	99.6	0.59 (0.48–0.73)	0.67 (0.56–0.79)	0.75 (0.63–0.89)	0.2261	<0.0001	44.4	99.5	0.66 (0.55–0.79)	0.87 (0.76–0.99)	0.80 (0.69–0.92)	0.0725	<0.0001
Grapes/grape juice	11.7	0.24	0.63 (0.53–0.75)	0.60 (0.50–0.72)	0.64 (0.52–0.77)	0.0087	<0.0001	14.2	0.30	0.83 (0.72–0.96)	0.70 (0.60–0.81)	0.68 (0.58–0.79)	<0.0001	0.0065
Anthocyanins from														
Grapes/grape juice	48.1	87.7	0.63 (0.53–0.75)	0.60 (0.50–0.72)	0.64 (0.52–0.77)	0.0087	<0.0001	51.7	88.7	0.81 (0.70–0.94)	0.69 (0.59–0.80)	0.67 (0.57–0.78)	<0.0001	0.0038
Strawberries	25.3	10.3	0.79 (0.66–0.94)	0.48 (0.39–0.59)	0.79 (0.65–0.96)	0.1853	<0.0001	24.0	9.40	0.83 (0.72–0.95)	0.69 (0.59–0.81)	0.66 (0.56–0.77)	<0.0001	0.0053
**Isoflavones from**														
Tofu	25.8	28.6	0.84 (0.70–1.02)	1.07 (0.89–1.29)	0.87 (0.71–1.07)	0.4473	0.0184	25.9	30.9	0.75 (0.64–0.87)	1.02 (0.88–1.19)	0.82 (0.70–0.97)	0.2771	<0.0001
Soybean paste ^g^	19.1	3.60	0.82 (0.68–0.98)	0.76 (0.63–0.91)	0.85 (0.70–1.03)	0.3299	0.0193	18.9	3.73	0.82 (0.70–0.96)	0.79 (0.68–0.92)	0.79 (0.68–0.92)	0.0218	0.0288
Cooked rice with beans	10.2	40.0	0.74 (0.62–0.89)			NA	NA	10.9	34.1	0.77 (0.67–0.89)			NA	NA
Multi–grain rice	9.72	0.75	0.51 (0.43–0.62)	0.83 (0.71–0.97)		0.0017	<0.0001	12.7	0.68	0.37 (0.30–0.45)	0.52 (0.45–0.6)	0.82 (0.72–0.94)	0.002	<0.0001
Soybean/soybean cooked in soy sauce	8.61	15.0	0.35 (0.26–0.49)	0.81 (0.69–0.94)	0.65 (0.55–0.77)	0.0008	<0.0001	8.79	19.7	0.39 (0.31–0.49)	0.80 (0.70–0.92)	0.69 (0.60–0.80)	0.0013	<0.0001
**Proanthocyanindins**														
Apple/apple juice	27.0	75.0	0.72 (0.60–0.86)	0.66 (0.55–0.80)	0.63 (0.52–0.76)	0.0005	0.0004	29.7	78.8	0.82 (0.71–0.94)	0.62 (0.53–0.72)	0.72 (0.62–0.85)	0.0055	<0.0001
Grapes/grape juice	17.1	14.9	0.63 (0.53–0.75)	0.60 (0.50–0.72)	0.63 (0.52–0.77)	0.0074	<0.0001	18.7	13.9	0.81 (0.70–0.94)	0.69 (0.59–0.80)	0.67 (0.58–0.78)	<0.0001	0.0039
Strawberries	13.3	5.52	0.79 (0.66–0.93)	0.48 (0.39–0.59)	0.79 (0.66–0.96)	0.1941	<0.0001	13.1	4.39	0.82 (0.71–0.94)	0.69 (0.59–0.81)	0.65 (0.55–0.76)	<0.0001	0.0041
Multi-grain rice	12.8	1.23	0.53 (0.44–0.64)	0.80 (0.68–0.93)		0.0006	<0.0001	13.5	0.64	0.36 (0.30–0.45)	0.51 (0.44–0.58)	0.86 (0.75–0.98)	0.0058	<0.0001

^a^ Major food sources: Foods with contents of 10% or more of flavonoids and specific flavonoids subclass intake or contribution to variation of 10% or more of flavonoids and specific flavonoids subclass intake. ^b^ Food items contributing to ≥10% of individual subclasses are shown. ^c^ Partial r^2^ from multiple stepwise regression with individual subclass as the dependent variable and individual content from each food item as the independent variable. ^d^ *p*-values for linear trends were obtained by treating the median value of each group as a continuous variable in multivariable model 1 adjusted for age (years), higher education level (≥12 years of schooling), regular exercise (≥3 times/week and ≥30 min/session), smoking (current/past/non-smokers for men and current/non-smokers for women), current drinkers (yes or no), body mass index (BMI), total energy intake (kcal/d), family history of hypertension (yes or no), menopausal status (yes or no for only women), and baseline blood pressures. ^e^ *p*-values for non-linear trends were obtained by comparing the deviance difference between the linear trend model on 1 degree of freedom (d.f.) and ℓ ordered categorical model on ℓ − 1 d.f. ^f^ Shepherd’s purse, beetroot, curled mallow, mugwort, outer leaves, etc. ^g^ soybean paste (*Doenjang*) soup, fast-fermented bean paste (*Chengguk-jang*), soybean paste (*Doenjang*), and *Ssamjang*.

## Data Availability

Data described in the manuscript will be made available upon request to the corresponding author.

## Supplementary Materials

The following supporting information can be downloaded at: https://www.mdpi.com/article/10.3390/nu15051186/s1, Table S1: Baseline characteristics of the study participants (n = 10,325); Table S2: Contribution of the seven flavonoid subclasses to flavonoid intake (n = 10,325); Table S3: Incidence rate ratio of hypertension by quartiles of dietary intake of flavonoids and their subclasses (n = 10,325); Table S4: Incidence rate ratio of hypertension by quartiles of dietary intake of 35 individual flavonoid compounds (n = 10,325); Figure S1: Timeline of study follow-up in KoGES_CAVAS cohort; Figure S2: Flowchart of study in KoGES_CAVAS cohort.
