# Supplementary material for: Prospective Associations between Cumulative Average Intake of Flavonoids and Hypertension Risk in the CArdioVascular Disease Association Study (CAVAS)
Source: Nutrients. 2023 Feb 27;15(5):1186. doi: 10.3390/nu15051186 (PMC10004779; doi:10.3390/nu15051186)
Supplement: Supplementary file 1 [file nutrients-15-01186-s001.zip › nutrients-2212984-supplementary.pdf]

Supplementary Table S1. Baseline characteristics of the study participants (n = 10,325)[a]

Supplementary Table S2. Contribution of the seven flavonoid subclasses to flavonoid intake (n = 10,325)

Supplementary Table S3. Incidence rate ratio of hypertension by quartiles of dietary intake of flavonoids and their subclasses (n = 10,325)

Supplementary Table S4. Incidence rate ratio of hypertension by quartiles of dietary intake of 35 individual flavonoid compounds (n = 10,325)

Supplementary Figure S1. Timeline of study follow-up in KoGES_CAVAS cohort

Supplementary Figure S2. Flowchart of study in KoGES_CAVAS cohort

Supplementary Table S1. Baseline characteristics of the study participants (n = 10,325)[a]

Characteristic	Total	Men	Women	*p*-value
N (%)	10,325	3,766 (36.5)	6,559 (63.5)	_
No. cases/ person-year	2,159 /53,678	854 /19,369	1,305 /34,308	
Age, y	56.9 ± 9.73	58.5 ± 9.73	55.9 ± 9.61	**<.0001**
Higher education[b]	31.6	38.3	27.7	**<.0001**
Regular exercise[c]	21.2	20.0	21.8	**0.0277**
Current smoker				
Never-smoker	70.9	27.3	95.9	**<.0001**
Past smoker	13.2	33.9	1.30	
Current smoker	16.0	38.8	2.85	
Menopausal status	70.1	_	70.1	_
Family history of hypertension	17.8	13.9	20.0	**<.0001**
Current drinker	42.1	62.6	30.3	**<.0001**
Alcohol consumption, ml/d	11.3 ± 35.9	26.8 ± 54.4	2.42 ± 10.9	**<.0001**
Body Mass index, kg/m^2	23.9 ± 3.00	23.7 ± 2.85	24.0 ± 3.08	**<.0001**
Waist circumference, cm	82.6 ± 8.66	84.5 ± 7.91	81.5 ± 8.88	**<.0001**
Total energy intake, kcal/d	1,579 ± 438	1,699 ± 445	1,509 ± 418	**<.0001**
Modified DASH scores[d]	17.4 ± 3.14	17.2 ± 3.18	17.5 ± 3.11	**0.0004**
**Total flavonoids, mg/d**	176 ± 164	161 ± 142	185 ± 175	_
Flavonols	18.1 ± 14.0	18.1 ± 13.5	18.0 ± 14.3	0.7977
Flavones	1.85 ± 1.28	1.73 ± 1.13	1.91 ± 1.36	**<.0001**
Flavanones	9.03 ± 10.9	7.11 ± 9.07	10.1 ± 11.7	**<.0001**
Flavan-3-ols	58.4 ± 112	55.7 ± 99.4	60.0 ± 118	**0.0461**
Anthocyanins	8.87 ± 10.8	7.25 ± 9.03	9.66 ± 11.7	**<.0001**
Isofavones	19.1 ± 15.3	19.7 ± 15.8	18.7 ± 15.0	**<.0001**
Proanthocyanins	60.7 ± 62.3	51.1 ± 51.7	66.2 ± 67.0	**<.0001**

[a] Values are expressed as mean ± standard deviation for continuous variables and percentages for categorical variables.
[b] Higher education level (≥12 years of schooling).
[c] Regular exercise (≥3 times/week and ≥30 min/session).
[d] Modified Dietary Approaches to Stop Hypertension (DASH) scores were calculated based on six out of eight components after excluding fruits and vegetables and modifying the 'Nuts/legumes' into 'Nuts' without legumes.

Supplementary Table S2. Contribution of the seven flavonoid subclasses to flavonoid intake (n = 10,325)

Ranking[a]	Subclasses	Men			Women		
		Median (IQR), mg/day	Contribution, %[b]	Variation, %[c]	Median (IQR), mg/day	Contribution, %	Variation, %
1	Proanthocyanindins	36.5 (19.7-64.2)	33.06	22.3	46.4 (24.3-85.8)	37.34	24.21
2	Flavan-3-ols	17.4 (6.36-52.7)	24.07	74.2	18.5 (7.06-52.6)	22.34	73.17
3	Isoflavones	15.1 (8.58-25.9)	16.92	1.82	14.2 (8.31-25.0)	14.58	0.7
4	Flavonols	15.0 (9.83-22.3)	15.13	1.00	14.5 (8.99-22.6)	13.03	0.12
5	Flavanones	4.58 (1.82-9.21)	4.80	0.53	6.79 (2.95-13.3)	6.01	0.11
6	Anthocyanins	4.60 (1.90-9.01)	4.57	0.14	6.19 (2.63-12.7)	5.32	0.36
7	Flavones	1.49 (0.97-2.16)	1.45	0.00	1.60 (1.02-2.43)	1.38	1.34
Total	Flavonoids	118 (69.3-206)	100%	100%	133 (75.8-236)	100%	100%

IQR: Interquartile range.

[a] Ranking of the contribution of individual subclasses to total flavonoid intake.

[b] Contribution to total flavonoid intake.

[c] Proportion of explained variation by the regression model.

Supplementary Table S3. Incidence rate ratio of hypertension by quartiles of dietary intake of flavonoids and their subclasses (n = 10,325)

Sensitivity analysis	Men (n = 3766)						Women (n = 6559)					
	Q1	Q2	Q3	Q4	$P_{linearity}$[a]	$P_{non-linearity}$[b]	Q1	Q2	Q3	Q4	$P_{linearity}$	$P_{non-linearity}$
**Total flavonoids**												
After censoring CVD and cancer	1 (ref)	0.66 (0.55-0.81)	0.71 (0.58-0.86)	0.84 (0.68-1.04)	0.9985	<0.0001	1 (ref)	0.74 (0.64-0.86)	0.81 (0.69-0.95)	0.89 (0.74-1.07)	0.8887	0.0002
Excluding cases within the first 2 year[c]	1 (ref)	0.69 (0.56-0.84)	0.67 (0.54-0.84)	0.82 (0.66-1.03)	0.7762	<0.0001	1 (ref)	0.79 (0.67-0.94)	0.83 (0.70-1.00)	0.82 (0.67-1.00)	0.2752	0.0350
Only non-users of antioxidant component supplement[e]	1 (ref)	0.67 (0.55-0.82)	0.67 (0.54-0.84)	0.81 (0.64-1.02)	0.6062	<0.0001	1 (ref)	0.67 (0.56-0.79)	0.82 (0.69-0.98)	0.85 (0.70-1.05)	0.8839	<0.0001
**Flavonols**												
After censoring CVD and cancer	1 (ref)	0.63 (0.52-0.76)	0.69 (0.57-0.84)	0.75 (0.61-0.93)	0.1510	<0.0001	1 (ref)	0.74 (0.63-0.86)	0.72 (0.61-0.84)	0.83 (0.69-0.98)	0.2808	<0.0001
Excluding cases within the first 2 year	1 (ref)	0.66 (0.54-0.81)	0.73 (0.60-0.90)	0.84 (0.68-1.05)	0.6960	0.0002	1 (ref)	0.74 (0.63-0.87)	0.69 (0.58-0.83)	0.76 (0.63-0.93)	0.0718	0.0003
Only non-users of antioxidant component supplement	1 (ref)	0.63 (0.52-0.78)	0.69 (0.56-0.85)	0.79 (0.63-0.99)	0.2915	<0.0001	1 (ref)	0.72 (0.61-0.85)	0.70 (0.59-0.84)	0.83 (0.68-1.01)	0.3114	<0.0001
**Flavones**												
After censoring CVD and cancer	1 (ref)	0.78 (0.64-0.94)	0.68 (0.55-0.83)	0.91 (0.73-1.13)	0.6824	0.0001	1 (ref)	0.93 (0.80-1.08)	0.79 (0.67-0.93)	0.96 (0.80-1.15)	0.6652	0.0074
Excluding cases within the first 2 year	1 (ref)	0.89 (0.73-1.09)	0.72 (0.58-0.90)	0.97 (0.77-1.24)	0.9303	0.0039	1 (ref)	0.91 (0.77-1.07)	0.76 (0.64-0.91)	0.86 (0.71-1.05)	0.1573	0.0320

Only non-users of antioxidant component supplement	1 (ref)	0.75 (0.62-0.92)	0.64 (0.51-0.80)	0.89 (0.70-1.12)	0.5147	<0.0001	1 (ref)	0.90 (0.76-1.06)	0.83 (0.70-1.00)	0.98 (0.80-1.20)	0.9252	0.0690
**Flavanones**												
After censoring CVD and cancer	1 (ref)	0.65 (0.54-0.79)	0.57 (0.47-0.69)	0.73 (0.60-0.89)	0.0955	<0.0001	1 (ref)	0.78 (0.67-0.90)	0.77 (0.66-0.89)	0.73 (0.62-0.86)	0.0033	0.0120
Excluding cases within the first 2 year	1 (ref)	0.68 (0.56-0.83)	0.58 (0.47-0.72)	0.75 (0.60-0.93)	0.1588	<0.0001	1 (ref)	0.82 (0.70-0.96)	0.80 (0.68-0.95)	0.72 (0.60-0.87)	0.0032	0.1700
Only non-users of antioxidant component supplement	1 (ref)	0.62 (0.51-0.75)	0.55 (0.45-0.68)	0.67 (0.54-0.83)	0.0201	<0.0001	1 (ref)	0.79 (0.67-0.93)	0.79 (0.66-0.93)	0.74 (0.61-0.90)	0.0129	0.0450
**Flavan-3ols**												
After censoring CVD and cancer	1 (ref)	0.64 (0.53-0.78)	0.69 (0.57-0.84)	0.77 (0.63-0.93)	0.8121	<0.0001	1 (ref)	0.82 (0.71-0.96)	0.89 (0.76-1.05)	0.86 (0.73-1.02)	0.5101	0.0450
Excluding cases within the first 2 year	1 (ref)	0.62 (0.50-0.76)	0.70 (0.57-0.86)	0.74 (0.60-0.91)	0.6609	<0.0001	1 (ref)	0.92 (0.78-1.08)	0.96 (0.81-1.15)	0.89 (0.74-1.07)	0.3214	0.5800
Only non-users of antioxidant component supplement	1 (ref)	0.65 (0.53-0.79)	0.67 (0.55-0.83)	0.71 (0.58-0.88)	0.2816	<0.0001	1 (ref)	0.79 (0.67-0.93)	0.90 (0.75-1.07)	0.86 (0.71-1.03)	0.5650	0.0210
**Anthocyanins**												
After censoring CVD and cancer	1 (ref)	0.73 (0.61-0.88)	0.62 (0.51-0.76)	0.72 (0.58-0.88)	0.0331	<0.0001	1 (ref)	0.76 (0.66-0.89)	0.72 (0.62-0.84)	0.74 (0.63-0.87)	0.0081	0.0008
Excluding cases within the first 2 year	1 (ref)	0.74 (0.61-0.89)	0.62 (0.50-0.76)	0.70 (0.56-0.87)	0.0222	0.0003	1 (ref)	0.79 (0.67-0.94)	0.78 (0.66-0.92)	0.71 (0.60-0.85)	0.0029	0.0530
Only non-users of antioxidant component supplement	1 (ref)	0.70 (0.58-0.85)	0.57 (0.46-0.70)	0.69 (0.55-0.86)	0.0196	<0.0001	1 (ref)	0.79 (0.66-0.93)	0.74 (0.62-0.88)	0.79 (0.66-0.94)	0.0597	0.0054
**Isoflavones**												
After censoring CVD and cancer	1 (ref)	0.76 (0.63-0.92)	0.62 (0.51-0.76)	0.76 (0.62-0.93)	0.0579	<0.0001	1 (ref)	0.76 (0.65-0.88)	0.68 (0.58-0.80)	0.81 (0.69-0.95)	0.1356	<0.0001
Excluding cases within the first 2 year	1 (ref)	0.79 (0.65-0.97)	0.67 (0.55-0.83)	0.83 (0.67-1.03)	0.3365	0.0013	1 (ref)	0.76 (0.65-0.91)	0.69 (0.58-0.83)	0.82 (0.68-0.98)	0.2417	0.00025
Only non-users of antioxidant component supplement	1 (ref)	0.74 (0.60-0.90)	0.59 (0.48-0.73)	0.74 (0.60-0.91)	0.0370	<0.0001	1 (ref)	0.73 (0.62-0.87)	0.67 (0.56-0.79)	0.77 (0.64-0.92)	0.0567	<0.0001
**Proanthocyanidins**												
After censoring CVD and cancer	1 (ref)	0.82 (0.68-0.98)	0.74 (0.61-0.90)	0.76 (0.61-0.94)	0.0487	0.0540	1 (ref)	0.79 (0.68-0.92)	0.71 (0.61-0.83)	0.73 (0.61-0.87)	0.0043	0.0029
Excluding cases within the first 2 year	1 (ref)	0.77 (0.63-0.94)	0.75 (0.61-0.92)	0.72 (0.57-0.91)	0.0345	0.0650	1 (ref)	0.81 (0.68-0.95)	0.74 (0.62-0.88)	0.68 (0.56-0.83)	0.0080	0.0710
Only non-users of antioxidant component supplement	1 (ref)	0.77 (0.63-0.93)	0.69 (0.56-0.86)	0.70 (0.56-0.88)	0.0135	0.0270	1 (ref)	0.77 (0.65-0.91)	0.70 (0.59-0.84)	0.78 (0.64-0.95)	0.0609	0.0013

DASH, Dietary Approaches to Stop Hypertension) score.

The multivariable model was adjusted for age (years), higher education level (≥12 years of schooling), regular exercise (≥3 times/week and ≥30 min/session), smoking (current/past/non-smokers for men and current/non-smokers for women), current drinkers (yes or no), body mass index (BMI), total energy intake (kcal/d), family history of hypertension (yes or no), menopausal status (yes or no for only women), and baseline blood pressures.

[a] *P* values for linear trends were obtained by treating the median value of each group as a continuous variable.

[b] *P* values for non-linear trends were obtained by Wald test to compare the deviance of the linear trend model to the deviance of the categorical model.

[c] Among participants excluding cases within the first 2 years after baseline (n = 3,568 for men, n = 6,224 for women)

[d] Among only non-users of antioxidant component supplements (n = 3,112 for men, n = 4,914 for women)

Supplementary Table S4. Incidence rate ratio of hypertension by quartiles of dietary intake of 35 individual flavonoid compounds (n = 10,325)

Flavonoids, mg/d	Men (n = 3766)						Women (n = 6559)					
	Q1	Q2	Q3	Q4	$P_{linearity}$[a]	$P_{non-linearity}$[b]	Q1	Q2	Q3	Q4	$P_{linearity}$[a]	$P_{non-linearity}$[b]
**Flavonols**												
Quercetin	1 (ref)	**0.72 (0.60-0.87)**	**0.69 (0.57-0.84)**	**0.77 (0.62-0.95)**	0.0851	**0.0009**	1 (ref)	**0.72 (0.62-0.83)**	**0.72 (0.62-0.84)**	**0.85 (0.72-1.00)**	0.4000	**<0.0001**
Kaempferol	1 (ref)	**0.70 (0.58-0.84)**	**0.69 (0.57-0.83)**	**0.81 (0.67-0.98)**	0.3972	**0.0001**	1 (ref)	0.86 (0.74-1.00)	**0.78 (0.67-0.92)**	0.86 (0.73-1.01)	0.2226	**0.0180**
Myricetin	1 (ref)	**0.72 (0.59-0.87)**	0.90 (0.75-1.09)	0.97 (0.80-1.18)	0.3774	**0.0012**	1 (ref)	**0.82 (0.71-0.95)**	**0.77 (0.66-0.89)**	0.92 (0.78-1.08)	0.8652	**0.0006**
Isorhamnetin	1 (ref)	**0.67 (0.56-0.80)**	**0.66 (0.55-0.79)**	**0.75 (0.62-0.90)**	0.0855	**<0.0001**	1 (ref)	**0.73 (0.63-0.85)**	**0.68 (0.58-0.79)**	**0.68 (0.58-0.80)**	**0.0002**	**0.0002**
**Flavones**												
Luteolin	1 (ref)	**0.68 (0.57-0.82)**	**0.66 (0.54-0.79)**	**0.71 (0.58-0.87)**	**0.0127**	**0.0002**	1 (ref)	**0.78 (0.67-0.90)**	**0.72 (0.62-0.84)**	0.89 (0.76-1.05)	0.5016	**<0.0001**
Apigenin	1 (ref)	**0.80 (0.67-0.96)**	**0.72 (0.59-0.88)**	0.99 (0.81-1.22)	0.4619	**0.0003**	1 (ref)	0.94 (0.81-1.09)	**0.81 (0.69-0.94)**	0.94 (0.79-1.12)	0.5063	**0.0220**
**Flavanones**												
Hesperetin	1 (ref)	**0.59 (0.50-0.71)**	**0.60 (0.50-0.72)**	**0.70 (0.58-0.84)**	0.1358	**<0.0001**	1 (ref)	**0.76 (0.66-0.88)**	**0.78 (0.67-0.91)**	**0.77 (0.66-0.91)**	0.0605	**0.0017**
Naringenin	1 (ref)	**0.59 (0.49-0.71)**	**0.57 (0.47-0.68)**	**0.69 (0.57-0.83)**	**0.0306**	**<0.0001**	*1 (ref)*	***0.78 (0.67-0.90)***	***0.75 (0.64-0.87)***	***0.73 (0.62-0.85)***	***0.0016***	**0.0074**
Eriodictyol	1 (ref)	**0.71 (0.60-0.86)**	**0.72 (0.59-0.87)**	**0.80 (0.66-0.98)**	0.7221	**0.0002**	1 (ref)	**0.83 (0.71-0.96)**	**0.83 (0.71-0.97)**	0.85 (0.72-1.01)	0.4341	**0.0220**
**Flavan-3ols**												
Catechin	1 (ref)	**0.79 (0.66-0.95)**	**0.71 (0.59-0.86)**	0.86 (0.70-1.05)	0.6054	**0.0015**	1 (ref)	0.87 (0.75-1.01)	**0.73 (0.62-0.86)**	**0.84 (0.70-0.99)**	0.1145	**0.0018**
Epicatechin	1 (ref)	**0.66 (0.55-0.80)**	**0.73 (0.61-0.88)**	0.86 (0.71-1.05)	0.8501	**<0.0001**	1 (ref)	0.76 (0.66-0.88)	0.72 (0.61-0.84)	0.92 (0.77-1.08)	0.8057	**<0.0001**
Epigallocatechin	1 (ref)	**0.74 (0.62-0.89)**	**0.71 (0.58-0.86)**	**0.76 (0.63-0.92)**	0.3267	**0.0006**	1 (ref)	**0.74 (0.64-0.86)**	0.88 (0.76-1.03)	**0.77 (0.65-0.90)**	0.0753	**0.0004**
Epicatechin gallate (ECG)	1 (ref)	**0.65 (0.54-0.78)**	**0.66 (0.55-0.80)**	**0.72 (0.60-0.86)**	0.3707	**<0.0001**	1 (ref)	**0.79 (0.68-0.91)**	0.90 (0.77-1.05)	**0.82 (0.70-0.96)**	0.2143	**0.0070**
Epigallocatechin gallate (EGCG)	1 (ref)	**0.64 (0.53-0.76)**	**0.61 (0.51-0.73)**	**0.67 (0.56-0.81)**	0.2157	**<0.0001**	1 (ref)	**0.77 (0.67-0.90)**	**0.85 (0.73-0.99)**	**0.76 (0.65-0.89)**	0.0532	**0.0030**
Gallocatechin	1 (ref)	**0.61 (0.51-0.73)**	**0.60 (0.50-0.72)**	**0.69 (0.57-0.82)**	0.3949	**<0.0001**	1 (ref)	**0.79 (0.68-0.91)**	0.86 (0.75-1.00)	**0.80 (0.68-0.94)**	0.1552	**0.0058**
Theaflavin	1 (ref)	**0.59 (0.48-0.73)**	**0.67 (0.56-0.79)**	**0.75 (0.63-0.89)**	0.2261	**<0.0001**	1 (ref)	**0.66 (0.55-0.79)**	0.87 (0.76-1.00)	**0.80 (0.69-0.92)**	0.0667	**<0.0001**
Thearubigin	1 (ref)	**0.59 (0.48-0.73)**	**0.67 (0.56-0.79)**	**0.75 (0.63-0.89)**	0.2261	**<0.0001**	1 (ref)	**0.66 (0.55-0.79)**	**0.87 (0.76-0.99)**	**0.80 (0.69-0.92)**	0.0723	**<0.0001**
Theaflavin 3'-gallate	1 (ref)	**0.59 (0.49-0.73)**	**0.67 (0.56-0.79)**	**0.75 (0.63-0.89)**	0.2261	**<0.0001**	1 (ref)	**0.66 (0.55-0.79)**	**0.87 (0.76-0.995)**	**0.80 (0.69-0.92)**	0.6667	**<0.0001**
Theaflavin 3-3'-digallate	1 (ref)	**0.59 (0.48-0.73)**	**0.67 (0.56-0.79)**	**0.75 (0.63-0.89)**	0.2261	**<0.0001**	1 (ref)	**0.66 (0.55-0.79)**	**0.87 (0.76-0.995)**	**0.80 (0.69-0.92)**	0.6667	**<0.0001**
**Anthocyanins**												
Cyanidin	1 (ref)	**0.80 (0.67-0.96)**	**0.65 (0.54-0.80)**	**0.72 (0.59-0.89)**	**0.0124**	**0.0023**	1 (ref)	**0.71 (0.61-0.83)**	**0.66 (0.57-0.77)**	**0.72 (0.60-0.85)**	**0.0102**	**<0.0001**
Delphinidin	1 (ref)	**0.65 (0.54-0.78)**	**0.60 (0.50-0.72)**	**0.69 (0.57-0.84)**	0.0588	**<0.0001**	1 (ref)	**0.78 (0.68-0.90)**	**0.67 (0.58-0.78)**	**0.68 (0.58-0.80)**	**<.0001**	**0.0002**
Malvidin	1 (ref)	**0.62 (0.52-0.74)**	**0.54 (0.45-0.65)**	**0.69 (0.57-0.84)**	0.1408	**<0.0001**	1 (ref)	***0.80 (0.69-0.92)***	***0.68 (0.59-0.80)***	***0.67 (0.57-0.78)***	***<.0001***	**0.0021**
Pelargonidin	1 (ref)	**0.76 (0.64-0.90)**	**0.56 (0.46-0.68)**	**0.74 (0.61-0.90)**	0.0881	**<0.0001**	1 (ref)	**0.80 (0.69-0.92)**	**0.77 (0.66-0.90)**	**0.66 (0.57-0.78)**	**<.0001**	**0.0230**
Peonidin	1 (ref)	**0.63 (0.53-0.75)**	**0.56 (0.47-0.68)**	**0.69 (0.57-0.83)**	0.0764	**<0.0001**	1 (ref)	**0.74 (0.64-0.85)**	**0.67 (0.57-0.78)**	**0.66 (0.57-0.77)**	**<.0001**	**0.0001**
Petunidin	1 (ref)	**0.64 (0.54-0.77)**	**0.58 (0.49-0.70)**	**0.68 (0.56-0.83)**	0.0586	**<0.0001**	1 (ref)	**0.76 (0.66-0.88)**	**0.69 (0.59-0.80)**	**0.66 (0.57-0.77)**	**<.0001**	**0.0005**
**Isoflavones**												
Daidzein	1 (ref)	**0.78 (0.65-0.94)**	**0.62 (0.51-0.74)**	**0.79 (0.65-0.96)**	0.0910	**<0.0001**	1 (ref)	**0.76 (0.65-0.89)**	**0.68 (0.58-0.79)**	**0.80 (0.68-0.93)**	0.0913	**<0.0001**
Genistein	1 (ref)	**0.73 (0.61-0.88)**	**0.61 (0.51-0.74)**	**0.76 (0.63-0.92)**	0.0734	**<0.0001**	1 (ref)	**0.78 (0.67-0.91)**	**0.68 (0.58-0.80)**	**0.80 (0.68-0.93)**	0.0584	**0.0001**
Glycitein	1 (ref)	**0.74 (0.62-0.89)**	**0.59 (0.49-0.71)**	**0.77 (0.64-0.94)**	0.1096	**<0.0001**	1 (ref)	**0.74 (0.63-0.86)**	**0.68 (0.58-0.80)**	**0.80 (0.68-0.93)**	0.1315	**<0.0001**
Biochanin A	1 (ref)	**0.73 (0.61-0.87)**	**0.60 (0.49-0.73)**	**0.74 (0.60-0.90)**	0.0868	**<0.0001**	1 (ref)	**0.77 (0.67-0.89)**	**0.68 (0.58-0.80)**	**0.72 (0.61-0.85)**	**0.0033**	**0.0002**

Formononetin	1 (ref)	0.90 (0.74-1.09)	0.87 (0.72-1.05)	1.13 (0.93-1.38)	0.1652	**0.0250**	1 (ref)	**0.74 (0.64-0.86)**	0.92 (0.79-1.06)	0.87 (0.75-1.01)	0.3491	**0.0006**
**Proanthocyanidins**												
Dimer	1 (ref)	**0.80 (0.67-0.96)**	**0.76 (0.62-0.91)**	**0.77 (0.62-0.95)**	0.0572	0.0530	1 (ref)	0.89 (0.76-1.03)	**0.74 (0.63-0.87)**	**0.83 (0.69-0.98)**	0.0613	**0.0062**
Trimer	1 (ref)	0.86 (0.72-1.03)	0.86 (0.71-1.04)	0.85 (0.68-1.05)	0.1979	0.3900	1 (ref)	**0.76 (0.65-0.88)**	**0.78 (0.67-0.91)**	**0.80 (0.67-0.96)**	0.0704	**0.0024**
4-6mers	1 (ref)	0.87 (0.73-1.05)	***0.76 (0.63-0.92)***	***0.77 (0.62-0.94)***	***0.0268***	0.1500	1 (ref)	***0.78 (0.67-0.90)***	***0.68 (0.58-0.79)***	***0.72 (0.61-0.86)***	***0.0049***	***0.0002***
7-10mers	1 (ref)	**0.76 (0.64-0.91)**	***0.59 (0.48-0.71)***	***0.69 (0.57-0.85)***	***0.0121***	**<0.0001**	1 (ref)	***0.76 (0.65-0.88)***	***0.71 (0.61-0.82)***	***0.69 (0.58-0.82)***	***0.0012***	***0.0006***
Polymer	1 (ref)	**0.69 (0.58-0.83)**	***0.67 (0.55-0.80)***	***0.67 (0.55-0.83)***	***0.0093***	**0.0003**	1 (ref)	***0.79 (0.68-0.91)***	***0.72 (0.62-0.84)***	***0.72 (0.61-0.85)***	***0.0016***	***0.0049***

Multivariable model was adjusted for age (years), higher education level (≥12 schooling years), regular exercise (≥3 times/week and ≥30 min/session), smoking (current/past/non-smokers for men and current/non-smokers for women), current drinkers (yes or no), body mass index (BMI), total energy intake (kcal/d), family history of hypertension (yes or no), menopausal status (yes or no for only women), and baseline blood pressures.

[a] *P* values for linear trends were obtained by treating the median value of each group as a continuous variable.

[b] *P* values for non-linear trends were obtained by comparing the deviance difference between linear trend model on 1 degree of freedom (d.f.) and ℓ ordered categorical model on $\ell - 1$ d.f.

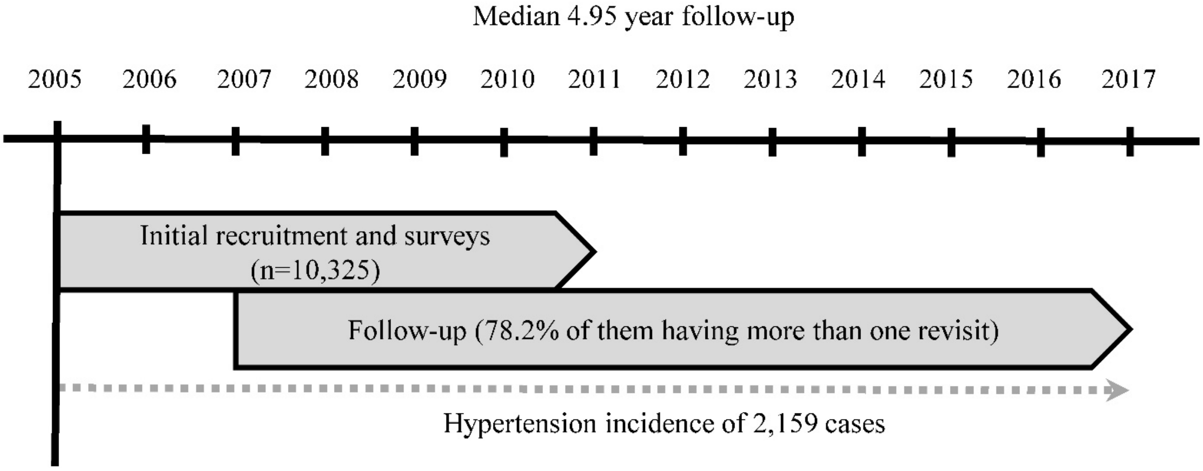

Supplementary Figure S1. Timeline of study follow-up in KoGES_CAVAS cohort

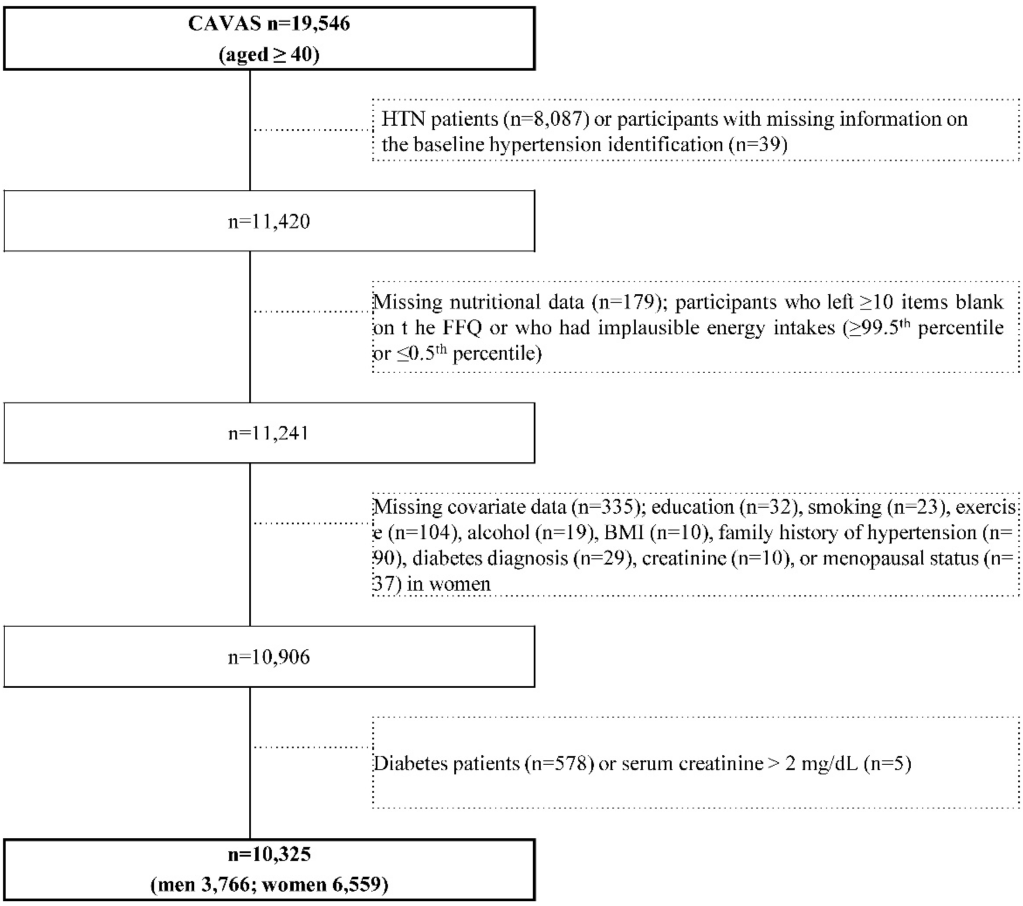

Supplementary Figure S2. Flowchart of study in KoGES_CAVAS cohort
